# Potential Diagnostic Biomarker Detection for Prostate Cancer Using Untargeted and Targeted Metabolomic Profiling

**DOI:** 10.3390/cimb45060320

**Published:** 2023-06-08

**Authors:** Diana Nitusca, Carmen Socaciu, Andreea Iulia Socaciu, Ioan Ovidiu Sirbu, Razvan Bardan, Alin Adrian Cumpanas, Edward Seclaman, Catalin Marian

**Affiliations:** 1 Department of Biochemistry and Pharmacology, Victor Babes University of Medicine and Pharmacy, Pta Eftimie Murgu Nr. 2, 300041 Timisoara, Romania; nitusca.diana@umft.ro (D.N.); ovidiu.sirbu@umft.ro (I.O.S.); eseclaman@umft.ro (E.S.); 2 Center for Complex Networks Science, Victor Babes University of Medicine and Pharmacy, Pta Eftimie Murgu Nr. 2, 300041 Timisoara, Romania; 3 BIODIATECH, Research Center for Applied Biotechnology in Diagnosis and Molecular Therapy, 400478 Cluj-Napoca, Romania; csocaciudac@gmail.com; 4 Department of Occupational Health, Iuliu Hateganu University of Medicine and Pharmacy, Str. Victor Babes Nr. 8, 400347 Cluj-Napoca, Romania; andreeaiso@gmail.com; 5 Department of Urology, Victor Babes University of Medicine and Pharmacy, Pta Eftimie Murgu Nr. 2, 300041 Timisoara, Romania; razvan.bardan@umft.ro (R.B.); cumpanas.alin@umft.ro (A.A.C.); 6 Urology Clinic, Timisoara Emergency County Hospital, 300723 Timisoara, Romania

**Keywords:** metabolomics, prostate cancer, biomarkers, diagnosis

## Abstract

Prostate cancer (PCa) remains one of the leading causes of cancer mortality in men worldwide, currently lacking specific, early detection and staging biomarkers. In this regard, modern research focuses efforts on the discovery of novel molecules that could represent potential future non-invasive biomarkers for the diagnosis of PCa, as well as therapeutic targets. Mounting evidence shows that cancer cells express an altered metabolism in their early stages, making metabolomics a promising tool for the discovery of altered pathways and potential biomarker molecules. In this study, we first performed untargeted metabolomic profiling on 48 PCa plasma samples and 23 healthy controls using ultra-high-performance liquid chromatography coupled with electrospray ionization quadrupole time-of-flight mass spectrometry (UHPLC-QTOF-[ESI+]-MS) for the discovery of metabolites with altered profiles. Secondly, we selected five molecules (L-proline, L-tryptophan, acetylcarnitine, lysophosphatidylcholine C18:2 and spermine) for the downstream targeted metabolomics and found out that all the molecules, regardless of the PCa stage, were decreased in the PCa plasma samples when compared to the controls, making them potential biomarkers for PCa detection. Moreover, spermine, acetylcarnitine and L-tryptophan had very high diagnostic accuracy, with AUC values of 0.992, 0.923 and 0.981, respectively. Consistent with other literature findings, these altered metabolites could represent future specific and non-invasive candidate biomarkers for PCa detection, which opens novel horizons in the field of metabolomics.

## 1. Introduction

One of the leading malignancies affecting men worldwide (of all races and ethnic groups) is prostate cancer (PCa). Globally, although this disease has a rapidly growing incidence (268,490 estimated new cases in 2022 alone in the US, according to the National Institutes of Health (NIH), it has a relatively high survival rate and favorable progression [1,2]. It typically affects middle-aged men (between 45 and 60 years), with risk factors including race, family history, genetic and environmental factors such as diet, smoking, obesity, chemical exposure, sexually-transmitted diseases and vasectomy [3].

The specific reasons behind the rise in PCa incidence over the years remain to be fully elucidated; modern research indicates that current diagnostic strategies are either highly invasive or lack proper specificity. This is the case for plasma prostate-specific antigen (PSA, ng/mL) screening, which, on the one hand, has helped reduce PCa-specific mortality over the last 30 years, but, on the other hand, has led to major overdiagnosis and over-treatment when used indiscriminately, due to its lack of specificity (being increased in other benign PCa-related diseases, such as prostatitis, benign prostatic hyperplasia, etc.) [4]. Other diagnostic methods include digital rectal examination (DRE), which is usually at risk for clinician subjectivity, magnetic resonance imaging (MRI) and the gold standard, transperineal prostate biopsy, which is highly invasive and leads to potential hospitalization due to infection, antibiotic use and the overall patient burden [5,6].

Therefore, in recent years, modern research has focused on the discovery of novel putative biomarkers that could improve the diagnosis and treatment of PCa, given the numerous publications dealing with this issue over the last decade. Such biomarkers belong to various classes of biological compounds, including proteins and other metabolites [7,8,9,10,11,12]. These molecules can now be easily identified and characterized (qualitatively and quantitatively) using a plethora of mass spectrometry-based techniques, generally designed as metabolomics approaches (referring to the scientific study of cellular processes involving small molecule metabolite profiles and their chemical fingerprints) [13]. In this regard, metabolomics is considered to represent the apogee of ”omic” (genomic, transcriptomic and proteomic) technologies and helps in finding a detailed understanding of various biochemical events inside cells and their relationship with each other. Different metabolomic studies have been reported in biomarker discovery on various diseases in recent years (including PCa and other cancers) as a new powerful technology and a dynamic field for global comprehension of biological systems. Commonly applied techniques in metabolomic analysis are mass spectrometry (MS)-based techniques, including gas chromatography–mass spectrometry (GC–MS), liquid chromatography–mass spectrometry (LC–MS) or magnetic resonance spectroscopy (MRS) [14,15,16].

Since cancer cells are characterized by altered metabolic pathways, the determination of low-molecular-weight metabolites in biological fluids (via liquid biopsy) could represent a novel, minimally invasive diagnostic method associated with high diagnostic potential and specificity, reduced invasiveness, increased compliance and a low overall burden for patients suffering from this disease [17].

Recently, it was found that free AA and lipid profiles vary depending on the type of cancer and its stage [18]. Examples of AA that were previously found to be dysregulated in PCa samples when compared to controls include alanine, arginine, uracil, glutamate (from tissue specimens) and valine, taurine and leucine (from both tissue and urine samples) [19]. In addition, one systematic review conducted by Kdadra et al. (2019) revealed that, in general, the vast majority of metabolites with altered profiles between PCa patients and controls belong to either the lipids class (lysophosphatidylcholine 18:2, decanoilcarnitine, stearate, docosadienoate), AAs (methionine, glutamine, isoleucine, arginine, leucine), amines (ethanolamine, sarcosine) and/or their derivatives [20]. Moreover, a laboratory-developed test called Prostarix (performed by the CLIA lab at Metabolon, Inc. and offered through Boswick Laboratories) is commercially available for use in the decision to perform initial or repeat biopsies for DRE-negative patients with moderately high PSA values. This test includes metabolomic profiling for sarcosine, glycine, alanine and glutamate, and it is performed from urine samples. However, Prostarix has not been approved by the FDA yet, and the urine samples need to be collected immediately following a vigorous DRE [21].

Therefore, in this study, we performed an untargeted metabolomics analysis on 48 plasma samples from diagnosed PCa patients and on 23 plasma samples from healthy controls using ultra-high-performance liquid chromatography coupled with electrospray ionization quadrupole time-of-flight-mass spectrometry (UHPLC-QTOF-[ESI+]-MS) to discover potential biomarkers for PCa detection. Next, based on this study and other literature findings, we selected five molecules for our downstream targeted metabolomics study: two free AAs (L-proline, L-tryptophan) and other endogenous molecules, such as acetylcarnitine, lysophosphatidylcholine C18:2 and spermine. Our aim was to identify potential biomarkers for a more minimally invasive diagnosis and staging of PCa.

## 2. Materials and Methods

### 2.1. Study Subjects and Clinical Data

This study comprised a total number of 71 subjects (48 PCa patients and 23 healthy controls). Blood was collected from each individual at the Urology Clinic of the Clinical Emergency County Hospital in Timisoara, Romania, following their agreement to participate in the study (all the subjects filled in a written IRB informed consent for the use of their biological specimens). The study was approved by the Ethics Committee of the participating institutions, in accordance with the 1964 Declaration of Helsinki and its later amendments, venous blood was collected in specific EDTA collection tubes; then, the plasma was separated and kept at −80 °C until further use. The patients were diagnosed with prostate adenocarcinoma following transrectal biopsies for histopathological diagnosis of PCa. The control volunteers had no prostate disease and normal PSA values (<4 ng/mL), verified by chemiluminescent microparticle immunoassay (Abbott Diagnostics, Chicago, IL, USA), and their blood samples were drawn at the same clinic.

### 2.2. Sample Preparation

A volume of 0.8 mL mix of methanol and acetonitrile (2:1 *v*/*v*) was added to 0.2 mL of blood plasma. The mixture was vortexed to precipitate the proteins, ultrasonicated for 5 min and kept for 24 h at −20 °C. The supernatant was collected after centrifugation at 12,500 rpm for 10 min (4 °C) and filtered through nylon filters (0.2 μm). Finally, the supernatant was placed in glass micro vials and transferred to the autosampler of the ultra-high-performance liquid chromatograph (UHPLC) before injection.

Quality control (QC) samples were also obtained by mixing the plasma samples to provide a representative “mean” sample and to validate the method. The QC samples were injected at the beginning and end of every 10th injection while analyzing the study samples in the UHPLC.

### 2.3. UHPLC-QTOF-ESI+-MS Analysis

The metabolic profiling was performed by ultra-high-performance liquid chromatography coupled with electrospray ionization quadrupole time-of-flight-mass spectrometry (UHPLC-QTOF-ESI+-MS) using a ThermoFisher Scientific UHPLC Ultimate 3000 instrument equipped with a quaternary pump, Dionex delivery system and MS detection equipment with MaXis Impact (Bruker Daltonics, Billerica, MA, USA). The metabolites were separated on an Acclaim C18 column (5 μm, 2.1 × 100 mm with a pore size of 30 nm (Thermo Scientific, Waltham, MA, USA) at 28 °C. The mobile phase consisted of 0.1% formic acid in water (A) and 0.1% formic acid in acetonitrile (B). The elution time was set at 20 min. The flow rate was set at 0.3 mL/min. The gradient for the plasma samples was: 90 to 85% A (0–3 min), 85–50% A (3–6 min), 50–30% (6–8 min), 30–5% (8–12 min) and, afterward, increased to 90% at 20 min. The volume of injected extract was 5 µL, and the column temperature was 25 °C. Several QC samples obtained from each group were used in parallel to calibrate the separations. Different volumes from a solution of 2 mg/mL Doxorubicin hydrochloride (MW = 580) were added in parallel to the QC samples as an internal standard (IS).

The ESI+-MS calibration was done with sodium formiate and the applied parameters were: capillary voltage of 3500 V, nebulizing gas pressure of 2.8 bar, drying gas flow of 12 L/min and drying temperature of 300 °C. The *m*/*z* values for the molecules to be separated were set between 50 and 600 Da. The control of the instrument and the data processing were done using the specific software TofControl 3.2, HyStar 3.2, Data Analysis 4.2 (Bruker, Daltonics, MA, USA) and Chromeleon, respectively.

### 2.4. Data Processing and Statistical Analysis

The Bruker software Data Analysis 4.2, attached to the instrument, was used to process the acquired data. By using the peak dissect algorithm, details of the separated molecules were obtained. By using the algorithm Find Molecular Features (FMF), we generated the first advanced bucket matrix, which included, for each *m*/*z* value, the retention time, the peak area, the peak intensity and the signal/noise (S/N) ratio. From the total ion chromatogram, using specific algorithms, total ion chromatograms (TICs) and base peak chromatograms (BPCs) were obtained. The number of separated molecules ranged between 1800 and 3000 in all the plasma samples.

The matrices representing the peak intensity = f(*m*/*z* value) for each sample were stored in an Excel file.

In the first step, we eliminated the molecules having retention times below 0.8 min (dead volume of the HPLC column), the molecules with S/N values < 5 (noise elimination), molecules with *m*/*z* values over 550 Da and minor molecules and residues with peak intensities under 1000 units. The number of molecules selected for the statistics decreased to 540 and the range of *m*/*z* values was between 100 and 550 nm.

In the second step, the alignment of common molecules (with the same *m*/*z* value) in all the samples, using the online software from www.bioinformatica.isa.cnr.it/NEAPOLIS, was performed, keeping for the final matrix the molecules common in more than 80% of the samples. The final matrices contained the *m*/*z* values versus the peak MS peak intensity for each molecule and each sample.

In some cases, no peak intensities were found, meaning that the molecule was not present, or it was present at intensities below 1000 (traces).

The Excel matrix (.xlsx) was converted to a .csv file, which was introduced in the Metaboanalyst 5.0 platform for multivariate and univariate analysis (https://www.metaboanalyst.ca/MetaboAnalyst/ModuleView.xhtml (accessed on 1 November 2022)).

The untargeted metabolomic analysis was performed by:

- Multivariate analysis comparing the control group with the whole patients’ group, based on the final matrix.csv for each type of sample. Discriminations between these two groups were represented by the fold change, volcano test, PatternHunter analysis, partial least squares discriminant analysis (PLS-DA), sparse PLSDA (sPLS-DA) and variable importance in the projection (VIP) values including cross-validation parameters. Then, the random forest-based prediction test was applied, and the calculations of *p*-values were performed by *t*-tests. The heat maps of correlations were also built. Finally, using the biomarker analysis, the receiver operating curves (ROCs) and the values of the areas under the ROC curves (AUCs) were obtained, and the *m*/*z* values were ranked according to their sensitivity/specificity.
- Univariate analysis, which allowed us to compare the subgroups P1-P4 with the control group. The statistical analysis was conducted for each type of sample using one-way ANOVA, PLS-DA and sPLS-DA score analysis, PatternHunter, random forest and heat maps.

The results were presented graphically, and the putative biomarkers of differentiation were identified. The identification of molecules, based on their *m*/*z* value and the retention time, was conducted in agreement with our own database and other international databases for metabolomics: the Human Metabolome Database (http://www.hmdb.ca, accessed on 1 November 2022), Lipid Maps (http://www.lipidmaps.org, accessed on 1 November 2022), PubChem (https://pubchem.ncbi.nlm.nih.gov, accessed on 1 November 2022) and the Heidelberg Database (https://www.msomics.com, accessed on 1 November 2022).

For the statistical analysis of targeted metabolomics, we selected the matrices which included the above-mentioned five metabolites (*m*/*z* values vs. MS peak intensity, as .csv file) and applied the Metaboanalyst 5.0 platform for multivariate and univariate analysis (https://www.metaboanalyst.ca, accessed on 1 November 2022).

Differences between the two groups (the control versus the PCa group) were first analyzed using the multivariate analysis by fold change, PCA and PLS-DA score plots including VIP values. Volcano plots were generated with the log2 fold change values and Bonferroni-adjusted *p* values. The value of *p* < 0.05 was defined as statistically significant.

To test the discriminatory capacity of each metabolite, we performed a receiver operating characteristic (ROC) analysis. For the AUC values higher than 0.8, the metabolite was considered to have a very high prediction of disease and can be used as a candidate biomarker for further study.

In the second step, the one-way ANOVA univariate analysis tested the discrimination between the controls and the subgroups of PCa patients (PI, PII, PIII and PIV). The PCA and PLS-DA score plots including VIP values, cross-validation parameters, as well the mean decrease accuracy scores by random forest analysis, were performed.

According to the data collected from the untargeted metabolomics, we targeted five specific molecules in the plasma, and their MS peak intensities were compared in the different groups of patients, e.g., between groups C and P. The mean values of peak intensity (PI) and their standard deviations SD were calculated for these specific biomarkers, selected by the untargeted metabolomics and in agreement with the recent literature data. For quantitative analysis, the calibration curves were built with pure standards.

### 2.5. Quantitative Evaluation

#### 2.5.1. Preparation of Calibration Solution and Quality Control (QC) Samples

The stock solutions of the five potential biomarkers, L-proline 10 mM, spermine 1 mM, acetylcarnitine 1 mM, L-Tryptophan 5 mM, lysophosphatidylcholine (LPC, 18:2) 2 mM, were dissolved in ultra-pure water. The stock solutions were successively diluted in the mix of methanol:acetonitrile 2:1 to obtain the series of working solutions at different concentration levels for external calibration. In parallel, volumes of 0.3 mL of QC deproteinated samples were spiked with different volumes of standard solutions.

#### 2.5.2. Method Validation

According to the “Guidance for Industry: Bioanalytical Method Validation” recommended by the US Food and Drug Administration (FDA), the UHPLC-QTOF-ESI+-MS method was validated to evaluate the linearity, specificity, precision, accuracy, the limit of detection (LOD) and the limit of quantification (LOQ). Two calibration curves were generated: an external standard calibration curve (1), made by diluting standard solutions in the mobile phase, and an internal standard curve (2), whose linearity was determined for the QC samples spiked with different volumes of standard solutions. The mean peak area of three replicate measurements at each concentration was calculated.

#### 2.5.3. Limit of Detection (LOD) and Limit of Quantification (LOQ)

The LOD was the lowest concentration of analyte in the test sample that could be reliably distinguished from zero to signal/noise ratio ≥ 10. The LOQ was the lowest concentration of analyte that could be determined with acceptable repeatability and trueness (signal/noise ratio ≥ 10 and SD values ≤ 40%).

### 2.6. Chemical Reagents

Pure standards of five targeted metabolites, namely L-proline, spermine, acetylcarnitine, L-tryptophan and lysophosphatidylcholine (LPC, 18:2) were purchased, as follows:

- L-proline (from Amino Acid Standard H product #20088, Thermo Scientific, Waltham, MA, USA). MW = 115.
- spermine (>97% product S3256, Sigma-Aldrich Chemie GmbH, St. Louis, MO, USA). MW = 202.
- o-acetyl-L-carnitine hydrochloride (J6153606; Alfa Aesar by Thermo Scientific, Waltham, MA, USA). MW = 203.
- L-tryptophan (>98.5% (T) (HPLC) (T0541;TCI Chemicals, Portland, OR, USA). MW = 204.
- lyso L-α-Lysophosphatidylcholine-LPC(18:2) from bovine brain (CAS nr. 9008-30-4) Sigma-Aldrich Chemie GmbH, St. Louis, MO, USA. MW = 519.

Other reagents such as HPLC-grade methanol, formic acid and acetonitrile were purchased from Sigma-Aldrich Chemie GmbH (St. Louis, MO, USA) and Thermo Fisher Scientific (Waltham, MA, USA). Ultrahigh-purity water was prepared by a Millipore-Q Water Purification System (Millipore, Darmstadt, Germany).

The instruments used in this study included a vortex mixer, Minicentrifuge Eppendorf (Thermo Fisher Scientific, Waltham, MA, USA) and UHPLC-QTOF-MS (Bruker GmbH, Bremen, Germany).

## 3. Results

### 3.1. Subjects’ Clinicopathological Data

A total number of 71 male subjects were included in this study, of whom 48 had histopathologically confirmed PCa (different stages, different PSA levels), and 23 were age-matched healthy controls. Their data are presented in Table 1.

Regarding demographics, the mean age of the patients was 63 years, while the mean age of the healthy controls was 54 years. More than half of the patients had PSA levels of 4–10 ng/mL (58.33%), while all the controls had PSA levels under 4 ng/mL (100%). The majority of tumors had Gleason scores of 7 or 8 (66.67%). We included in our study patients with different AJCC stages (I to IV), with the vast majority (81.25%) having either stage 2 or 3.

### 3.2. Untargeted Metabolomic Profiling

#### 3.2.1. Metabolite Identification

Following the discovery phase of our study, via the untargeted plasma profiling, we retrieved a total number of 296 molecules with molecular weights below 550 Da. These molecules were subsequently identified with putative names using different international databases, such as the Human Metabolome Database (http://www.hmdb.ca, accessed on 1 November 2022), Lipid Maps (http://www.lipidmaps.org, accessed on 1 November 2022), PubChem (https://pubchem.ncbi.nlm.nih.gov, accessed on 1 November 2022) and Heidelberg Database (https://www.msomics.com, accessed on 1 November 2022) and are shown in Table S1 (Supplementary Material). The vast majority of the metabolites identified were either AAs (such as L-proline, L-tryptophan, L-valine, L-threonine, L-aspartic acid, ornithine, L-lysine, L-glutamic acid, etc.) or products derived from AA metabolism. Other metabolites were amines (spermine, glutamine, serotonin) or, in general, nitrogen-containing compounds, while a substantial part was made of metabolites derived from lipid metabolism (phospholipids, lysophospholipids and fatty acids). A small part was metabolites of nucleotide structure, such as uridine, guanosine and inosine. This preliminary data is consistent with other literature findings regarding free AAs and lipid profiles.

#### 3.2.2. Multivariate Untargeted Metabolomics Statistical Analysis of the 71 Plasma Samples

**Figure 1:**
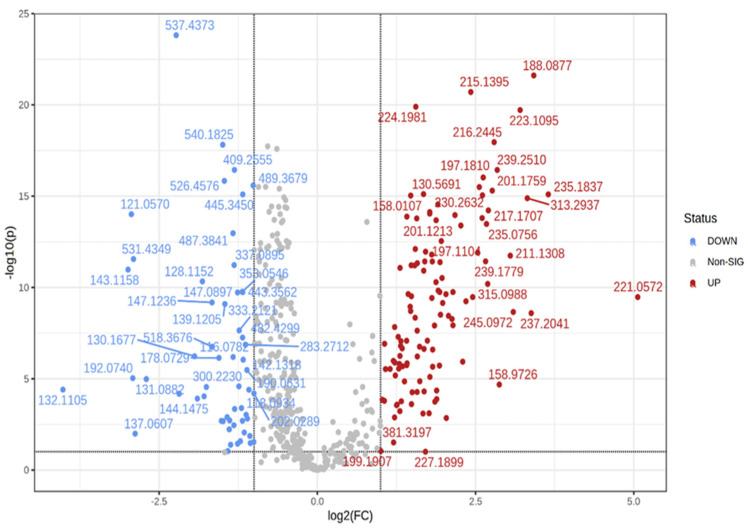
Volcano plot: red-marked *m*/*z* values are decreased in the patients’ group compared to the control group; blue-marked *m*/*z* values are increased in the patients’ group compared to the control group.

Following identification, we performed the statistical analysis. Volcano plots were generated to show the significant molecules which are increased or decreased between the groups: controls, n = 23, and patients, n = 48. The volcano plot (Figure 1) shows the molecules (*m*/*z* values) that have significantly lower or higher intensities in the patients’ group when compared to the control group.

**Figure 2:**
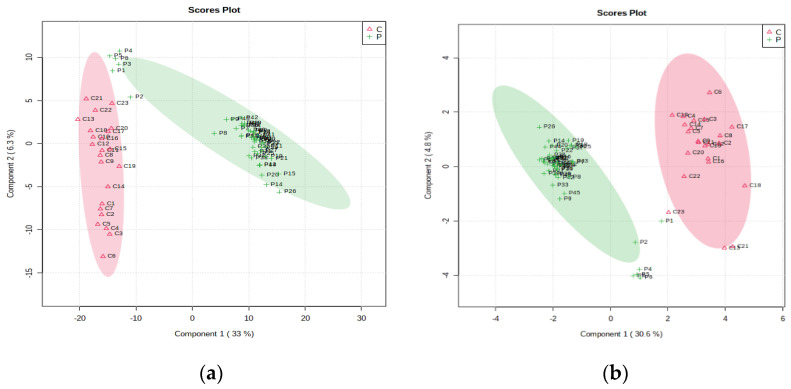
(**a**, **left**) PLS-DA plot with a covariance of 39.3% and (**b**, **right**) sPLS-DA plot with a covariance of 35.2% showing the discrimination between patient and control groups.

Next, a discrimination analysis was performed, where the partial least squares-discriminant analysis (PLS-DA) and sparse PLS-DA plots were generated. In general, they showed a good differentiation of metabolic patterns between the two groups (patients and controls). The sparse PLS-DA (sPLS-DA) algorithm reduces the number of feature variables (metabolites) and shows a more robust and easy-to-interpret model. One can control the “sparseness” of the model by adjusting the number of components in the model and the number of variables within each component (Figure 2a,b, respectively).

Then, we performed a biomarker analysis which included the analysis of the receiver operating characteristic (ROC) curve with the corresponding area under the curve values (AUC), and the metabolites were ranked based on the AUC values. The most representative molecules to discriminate between the control group and the patients’group, and to be considered putative biomarkers (with AUC values higher than 0.7), are shown in Table 2.

**Figure 3:**
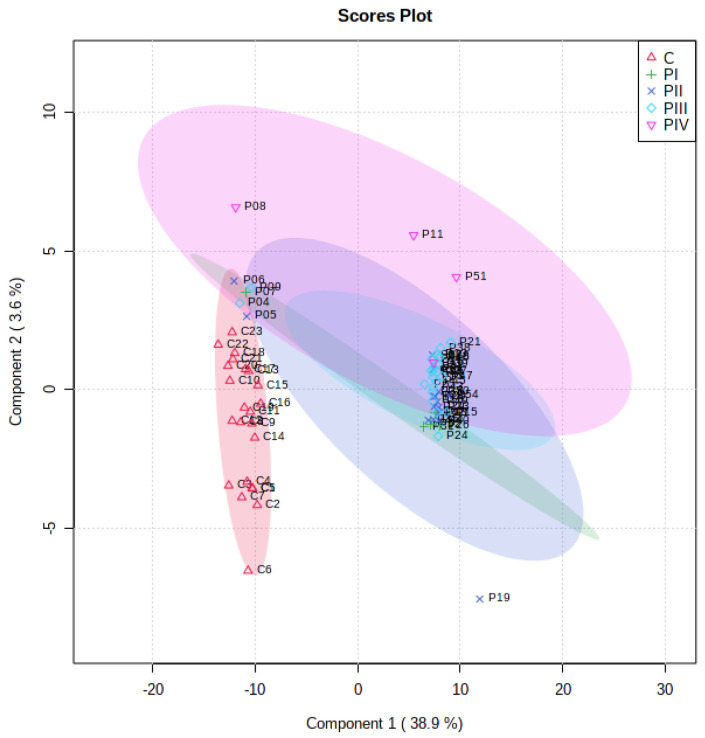
PLS-DA score plots showing the differences between controls and patients (subgroups PI-PIV).

In addition, a discrimination analysis was also performed for the patients’ group, according to their AJCC stage (I-IV), and for the controls, and the PLS-DA plot which resulted from the ANOVA analysis can be seen in Figure 3.

**Figure 4:**
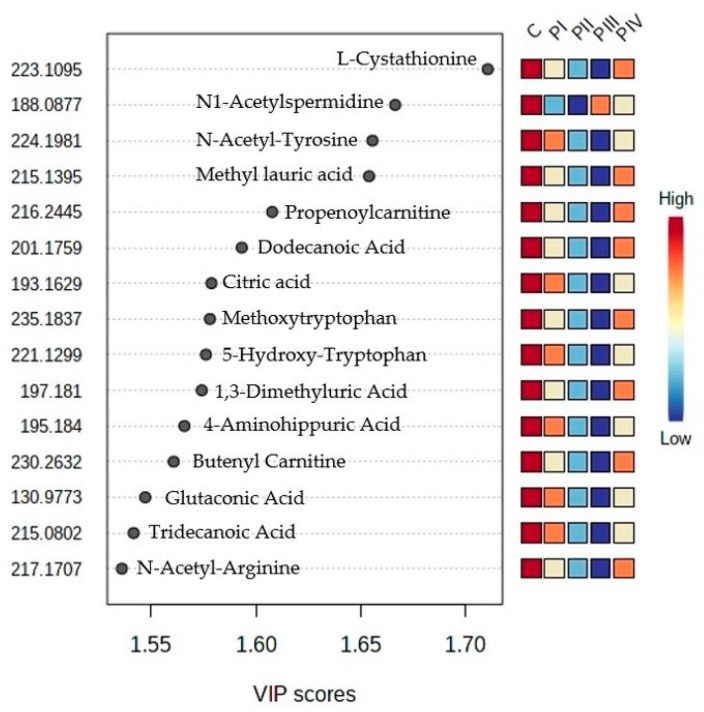
Ranking of top 15 molecules with higher VIP scores, according to PLS-DA analysis (their identification can be made by using Table S1).

From the PLS-DA analysis, the VIP scores were calculated and the ranking of the first 15 molecules to be considered responsible for the discrimination is presented in Figure 4.

### 3.3. Targeted Metabolomic Profiling

According to our previous untargeted analysis and consistent with other literature findings, we selected five different molecules (L-proline, spermine, acetylcarnitine, L-tryptophan and lysophosphatidylcholine LPC 18:2) for our downstream targeted study. Even though these molecules were not generally among the top-ranked metabolites found in our untargeted study, we chose them based on the available literature findings regarding the three main metabolisms affected in PCa: AAs, phosphatidylcholines and carnitines.

#### 3.3.1. Calibrations and Validation Parameters

The determination of linear ranges (calibration curves and equations including R^2^ values), the limit of detection (LOD) and the limit of quantification (LOQ) of each standard are given in Table 3. The correlation coefficients (R^2^) were higher than 0.898 for all the standards in their linear range, showing good linear relationships within linear ranges. All the LOD values were in the range of 0.3–4 μM, and the LOQ values were in the range of 0.9–5.5 μM.

The validation of the LC–MS method for the quantitative evaluation of the metabolites was conducted using controlled additions of the internal standard (doxorubicin—DOXO) and each of the five pure standards to the quality control (QC) extracts.

To the same volume of QC extracts (0.3 mL), we added 0.2 mL of the five standard solutions (50 μM L-proline, 5 μM spermine, acetylcarnitine or LPC and 20 μM L-tryptophan) and of the internal standard (DOXO 2 mg/mL = 3.44 μM) with known concentrations of each metabolite. Table 4 shows the initial concentrations of metabolites after mixing with the QC extract and the measured concentrations after the LC–MS analysis. The recovery percentage was calculated as a measure of the method’s reproducibility.

#### 3.3.2. Quantitative Determination

**Figure 5:**
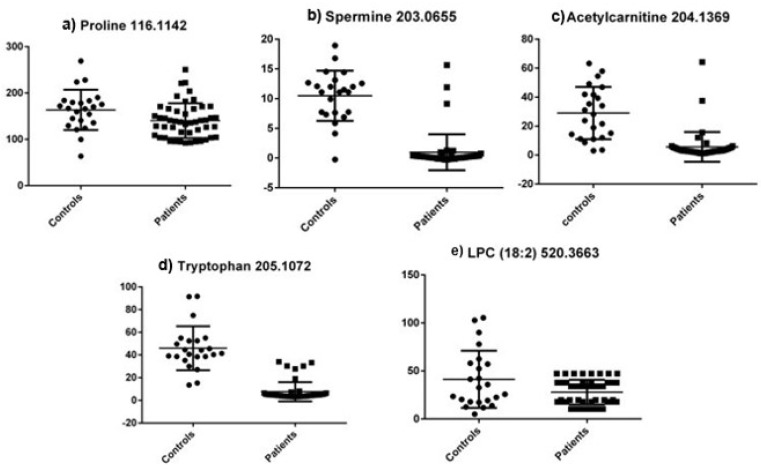
Metabolite concentrations (μM) between all patients and controls for the five selected molecules in the targeted analysis.

Unpaired *t*-tests showed that there were statistically significant differences between the patients (all stages) and controls for all five molecules analyzed. Spermine, acetylcarnitine and L-tryptophan showed *p* values below 0.0001, while L-proline and LPC 18:2 had *p* values of 0.02 and 0.01, respectively. All five molecules had significantly lower concentrations in the patients’ group (all stages) when compared to the control group, as seen in Figure 5.

Next, we performed ROC curve analyses for the aforementioned molecules, and the obtained data can be seen in Table 5.

**Figure 6:**
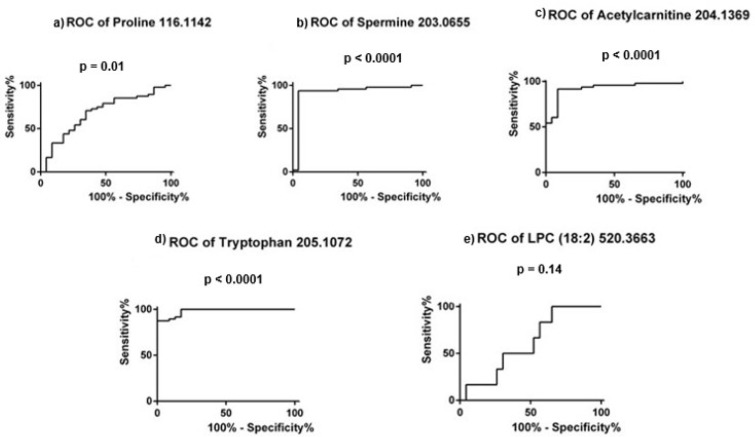
ROC curves for the five molecules selected in the targeted analysis.

According to this data, the molecule with the highest diagnostic potential is represented by L-tryptophan (AUC value of 0.981), suggesting very good diagnostic potential for PCa detection. The same is true for acetylcarnitine and spermine (AUC values of 0.923 and 0.922, respectively), while L-proline and LPC 18:2 showed only a moderate diagnostic value, both having AUC values below 0.7. The ROC curves for these five molecules can be seen in Figure 6.

The quantitative evaluation, based on the curve equations for each of the five biomarkers, is presented in Table 6.

All the metabolites had decreased levels in the patients’ group, regardless of their stage, when compared to the control group. For L-proline, we observed a gradual decrease in concentration from stage I to stage III, which slightly increased in stage IV. In addition, spermine levels decreased more than 10 times in the patients’ group (all stages) compared to the control group, having the most significant change of all metabolites. For acetylcarnitine, there was a gradual increase in concentration, which was directly proportional to stages II-IV, while the reverse occurred for L-tryptophan (stages I-III), which had a similar profile to L-proline.

In addition, to evaluate the staging potential of these five molecules, ordinary one-way ANOVA tests were performed on the values of concentration (μM) for the controls and for the patients grouped according to their AJCC stage (I, II, III and IV). Statistically significant differences were observed for spermine, acetylcarnitine and L-tryptophan (*p* < 0.0001), but not for L-proline (*p* = 0.209) or LPC 18:2 (*p* = 0.159).

## 4. Discussion

In the present study, we performed an untargeted and targeted metabolomic analysis of human plasma samples from PCa-diagnosed patients compared to healthy controls using UHPLC–MS. The aim was to discover and validate potential biomarkers for the early detection and staging of this urologic malignancy in a minimally invasive fashion (from plasma samples). In the targeted analysis, we performed calibrations and validation analyses to evaluate the concentrations and the changes in the levels of the five potential biomarkers targeted: L-proline, L-tryptophan, spermine, acetylcarnitine and lysophosphatidylcholine (18:2). We found out that, regardless of the PCa stage, all the selected metabolites were significantly decreased in the PCa plasma samples when compared to the healthy controls, making these molecules potential future biomarkers for PCa diagnosis.

In this regard, spermine is a polyamine involved in cellular metabolism, having ornithine AA as a precursor. Our findings regarding decreased levels of L-proline in PCa patients’ plasma samples vs. healthy controls are consistent with other literature findings, as Bentrad et al. (2019) described that the concentration of spermine was 7–34 times lower in PCa samples when compared to healthy individuals and even to patients with benign prostate hyperplasia [22]. In contrast, one study proposed spermine (among other metabolites) as a potential biomarker in distinguishing aggressive from indolent PCa, as spermine levels were decreased in the aggressive phenotype [23].

Acetylcarnitine, another potential biomarker that we found to be decreased in PCa plasma in our study, is an endogenous acetylated form of L-carnitine, a source that releases (by esterase hydrolysis) L-carnitine, which is known to be involved in the transport of fatty acids into the mitochondria for β-oxidation and ATP generation. In PCa, in particular, acetylcarnitine was shown to downregulate different pathways involved in angiogenesis (such as VEGF and CXCL8) and invasion (via the downregulation of CXCR4/CXCL12 and MMP-9), playing, therefore, a protective role against PCa development. The same study conducted by Baci et al. (2019) found that acetylcarnitine induces apoptosis of PCa cells, reduced cancer cell proliferation, and halted proinflammatory cytokines and chemokines production, such as TNF-α, IFN-γ and CCL2, CXCL12 and receptor CXCR4, respectively, therefore altering migration, invasion and adhesion processes. The authors, therefore, propose acetylcarnitine as a novel repurposed dietary supplement for chemoprevention in PCa [24].

L-tryptophan is an essential α-AA with an indole side chain, used in the biosynthesis of proteins. In PCa, in our study, we found out that L-tryptophan was significantly decreased in all the patients’ samples compared to the controls, resulting in the highest diagnostic accuracy (AUC value of 0.981). One study revealed that aromatic AAs (including L-tryptophan) have altered metabolisms and play harmonizing roles in PCa and that their modifications could be used as potential biomarkers in the early detection of this disease [25].

Another AA that we chose to investigate in our targeted metabolomic profiling is L-proline, a proteinogenic, secondary, non-essential AA, synthesized endogenously from L-glutamate. It is generally used in the biosynthesis of proteins, although it does not contain the free amino group, NH2. However, the exact role of L-proline metabolism in PCa is yet to be fully elucidated. Nonetheless, a very recent study (2022) proposes prostate-specific membrane antigen (PSMA) as a promising future therapeutic target against PCa, as it demonstrates that this protein modulates PCa progression by regulating arginine and L-proline biosynthesis (its depletion promoted the synthesis of these two AA and inhibited androgen receptor expression) [26].

In addition, lysophosphatidylcholines are minor phospholipids from cell membranes and are found in blood plasma (8–12%), including different acyl groups from saturated or unsaturated fatty acids. In this regard, Li et al. (2021) demonstrated that high LPC levels in urine are associated with PCa development, therefore proposing LPCs as novel biomarkers for the detection of this malignancy [27]. Moreover, Zhou et al. (2012) revealed that PCa progression is positively correlated with the expression level of the enzyme that catalyzes the remodeling of phosphatidylcholine (PC) reaction, namely lysophosphatidylcholine acyltransferase 1 (LPCAT1), and this correlation was independent of age, race and PSA levels of PCa patients, proposing LPCAT1 and LPC as novel biomarkers for PCa detection [28].

Moreover, significant associations between the lipid profile and malignancy were validated, and the phospholipid composition was characteristically altered in the tissues of patients who responded to androgen receptor inhibition. In this regard, a study of 40 prostate tissues and 40 healthy controls using two imaging methods showed, following the analysis of phosphatidylcholine, lysophosphatidylcholine, sphingomyelin and phosphatidylethanolamine classes, that prostate tumors are correlated with increased fatty acid synthesis and lipid oxidation. Phosphatidylcholine variants PC 16:0/16:1, PC 16:0/18:2, PC 18:0/22:5, PC 18:1/18:2, PC 18:1/20:0 and PC 18:1/20:4 showed the highest discriminatory power between the two tested groups, suggesting that lipidomics may represent an alternative future diagnostic strategy for PCa [29].

Nonetheless, it is clear that no single analytical method can accommodate the chemical diversity of the entire metabolome; thus, a multi-platform approach can provide a more comprehensive understanding of metabolic changes. Therefore, the metabolomic profile obtained by combining the MS approach with UHPLC techniques could obtain, with greater accuracy, more precise information regarding the mechanisms of PCa and could help in the much earlier detection of cancer, given the fact that the alteration of the cellular metabolism occurs in the early stages of the tumorigenesis process.

Taken together, our findings suggest that molecules belonging to the AA, lipid and polyamine classes might represent novel candidate biomarkers for PCa early detection and staging, using minimally invasive, *bona fide* tools such as metabolomics (and/or lipidomics).

Our study has some limitations, however, that mainly arise from the small population size used in this research (71 subjects). Another important characteristic that could not be adjusted for with the available blood samples from our participants was the 9-year age mismatch between the patients and the controls, which is known to possibly affect metabolite levels. In addition, our analyses do not adjust for other covariates, due to the limited information available regarding the participants (no information on smoking, history of diabetes, and BMI). Moreover, the ROC analyses from the targeted profiling were performed on the same population, as additional blood samples from a separate cohort were not available for this study. Another limitation of our study arises from the fact that, currently, there is no available scientific background to support our hypotheses. At the moment, there are no metabolomic case-control studies performed on acetylcarnitine, L-tryptophan, L-proline or LPC (18:2) in relation to PCa diagnosis and staging alone, in spite of the fact that their metabolism has been studied in tumorigenesis, in general, and in other human diseases [30,31,32,33,34]. Therefore, assessing their true diagnostic value as biomarkers for PCa detection and staging remains an issue in dire need of further extensive research in order to avoid providing a false diagnosis. Hence, our results are relatively preliminary and should be viewed in the larger context of biomarker discovery for PCa.

## Figures and Tables

**Table 1:** Characteristics of the subjects included in the present study.

Characteristics	N = 48 (Patients)	%	N = 23 (Controls)	%
Age (mean ± SD)	63 (±5.49)		54 (±1.29)	
Prostate-specific antigen—PSA (ng/mL)				
<4	0	0.00	23	100.00
4–10	28	58.33	0	0.00
>10	20	41.66	0	0.00
Prostatic volume (mL) mean ± SD	44 (±19.83)			
PSA density (ng/mL^2^) mean ± SD	0.28 (±0.15)			
Gleason score				
6	14	29.17		
7–8	32	66.67		
9	2	4.16		
AJCC stage				
I	5	4.16		
II-III	39	87.5		
IV	4	8.33		

**Table 2:** The ranking of the first molecules based on AUC values higher than 0.7.

*m*/*z*	AUC	*p*-Value	Identification
188.0877	0.9973	2.43 × 10^−22^	N1-Acetylspermidine
537.4373	0.9955	1.52 × 10^−24^	Stearyl Stearate & Isomers
335.298	0.9873	3.07 × 10^−16^	Docosatrienoic Acid C22:3
326.301	0.9846	1.86 × 10^−18^	N-Myristoyl Proline
215.1395	0.9828	1.973 × 10^−21^	Methyl lauric acid
540.1825	0.9828	1.525 × 10^−18^	Hexacosanoyl Carnitine
223.1095	0.9792	1.92 × 10^−20^	L-Cystathionine
489.3679	0.9792	2.59 × 10^−16^	Cytidine 5’-Diphosphocholine
331.0208	0.9774	2.56 × 10^−18^	Deoxycorticosterone
300.223	0.9765	2.66 × 10^−5^	D-Sphingosine
526.4576	0.9728	1.45 × 10^−16^	LPS(18:0)
239.251	0.9701	3.66 × 10^−17^	5-Oxo-Tetradecadienoic Acid (C14:2)
409.2555	0.9701	3.64 × 10^−17^	Ursocholic/Muricholic Acid Acid
205.1072	0.9692	3.16 × 10^−16^	L-Tryptophan
227.1899	0.9692	0.099	Carnosine
419.2139	0.9683	7.21 × 10^−15^	Palmitoyl Glucuronide
181.9605	0.9674	3.11 × 10^−16^	Tyrosine
224.1981	0.9674	1.26 × 10^−20^	N-Acetyl-Tyrosine

**Table 3:** Validation parameters (linear range, curve equation, the correlation coefficients (R^2^), limit of detection (LOD) and limit of quantification (LOQ) for each of the five molecules selected as potential biomarkers.

Molecule (*m*/*z*)	Linear Range μM	Curve Equation	R^2^	LOD μM	LOQ μM
L-proline (116.1142)	10–100	y = 11290x − 119623	0.898	4.0	5.5
Spermine (203.0655)	0.5–5	y = 498406x + 51799	0.997	0.1	0.3
Acetylcarnitine (204.1369)	1–5	y = 36813x − 3879.2	0.994	0.2	0.8
L-tryptophan (205.1072)	4–40	y = 19944x − 7756	0.998	0.8	1.0
LPC 18:2 (520.3663)	1–10	y = 172161x + 91874	0.965	0.3	0.9

**Table 4:** The recovery percentage (%) was calculated from the measured concentrations of internal standard (IS) and each metabolite (pure standard) compared to their initial concentrations, after addition to QC extract.

Metabolite	Initial Concentration (mM)	Measured Concentration (mM)	Recovery (%)
L-proline	20	17.5	87.5
Spermine	2	1.82	91.0
Acetylcarnitine	2	1.88	94.0
L-tryptophan	8	7.55	94.3
LPC(18:2)	2	1.85	92.5
IS (DOXO)	1.4	1.25	89.3

**Table 5:** ROC curve analysis for the molecules selected in the targeted metabolomic profiling.

Metabolite	*m*/*z*	AUC Value	95% CI	*p* Value
L-proline	116.1142	0.682	0.548–0.816	0.01
Spermine	203.0655	0.922	0.832–1.01	<0.0001
Acetylcarnitine	204.1369	0.923	0.855–0.991	<0.0001
L-tryptophan	205.1072	0.981	0.957–1.005	<0.0001
LPC (18:2)	520.3663	0.608	0.453–0.764	0.140

**Table 6:** The mean values of concentrations (μM) of the five potential biomarkers targeted in this study for the control group, patients’ group (all) and subgroups, PI, PII, PIII and PIV (according to their AJCC stage). The percentage of standard deviations (SD, %) for each group is also presented SD (%) = 100 × SD/mean value).

Molecule	Controls		Patients (All Stages)		PI		PII		PIII		PIV	
	Mean (μM)	SD (%)	Mean (μM)	SD (%)	Mean (μM)	SD (%)	Mean (μM)	SD (%)	Mean (μM)	SD (%)	Mean (μM)	SD (%)
L-proline	163.580	43.45	140.36	37.57	152.08	24.57	134.93	30.98	140.36	45.68	147.46	27.59
Spermine	10.484	4.22	0.98	3.04	3.05	7.05	0.06	0.37	1.19	2.99	0.87	0.10
Acetylcarnitine	29.020	18.03	5.65	10.27	4.10	4.56	3.49	1.69	6.29	12.98	12.53	16.70
L-tryptophan	45.908	19.39	7.63	8.45	9.67	11.58	7.60	9.45	6.16	5.67	13.68	13.65
LPC (18:2)	41.274	29.77	27.93	12.93	28.18	12.34	28.34	13.95	27.09	12.90	30.77	14.24

## Data Availability

All data is available in the manuscript.

## Supplementary Materials

The following supporting information can be downloaded at: https://www.mdpi.com/article/10.3390/cimb45060320/s1, Table S1. Metabolite identification with putative names via HMDB, PubChem, Lipid Maps and Heidelberg databases.
