# Supplementary material for: Potential Diagnostic Biomarker Detection for Prostate Cancer Using Untargeted and Targeted Metabolomic Profiling
Source: Curr Issues Mol Biol. 2023 Jun 8;45(6):5036–51. doi: 10.3390/cimb45060320 (PMC10296965; doi:10.3390/cimb45060320)
Supplement: Supplementary file 1 [file cimb-45-00320-s001.zip › cimb-2394614-supplementary.pdf]

Table S1. Metabolites identification with putative names via HMDB, PubChem, Lipid Maps and Heidelberg databases.

No	m/z	Identification
1.	103.0459	Acetoacetic Acid
2.	104.1132	Gama Aminobutiric Acid (GABA)
3.	105.0391	Hydroxypyruvic Acid
4.	107.0870	Glyceric Acid
5.	109.1077	Quinone
6.	110.0155	Hypotaurine
7.	110.0775	2-Aminophenol
8.	111.1236	Hydroquinone
9.	112.9022	Histamine
10.	113.0660	Uracil
11.	113.9705	Thiosulphate
12.	114.0982	Creatinina
13.	114.8996	5,6-Dihydrouracil
14.	116.0782	Caproic Acid
15.	**116.1142**	**Proline**
16.	117.0946	Methyl Acetoacetate
17.	118.0934	Valine
18.	120.0880	Threonine
19.	121.0570	Purine
20.	121.1086	Deoxythreonic Acid
21.	123.0857	Nicotinamide
22.	123.1245	Erythritol
23.	125.1044	4-Methylcatechol
24.	125.9940	Taurine
25.	127.0224	4-Imidazoleacetic Acid
26.	127.0804	Thymine
27.	129.1354	Dihydrothymine
28.	130.0198	5-Oxo-D-Proline
29.	130.0937	Glutaconic Acid
30.	130.1397	N-Methyl-L-Proline
31.	130.1677	Pyroglutamic Acid
32.	130.5691	Pipecolic Acid ((D-Homoproline))
33.	130.9773	Glutaconic Acid
34.	131.1148	N-Acetylputrescine
35.	132.1105	Leucine/Isoleucine
36.	133.0588	Asparagina
37.	133.1092	Ornitine
38.	134.0692	Aspartic Acid

39.	135.0187	Deoxyribose
40.	135.1250	L-Malic Acid
41.	136.0468	Adenine
42.	136.1170	Homocysteine
43.	137.0607	Hipoxanthine
44.	137.1411	Phenylacetic Acid
45.	138.0970	Tyramine
46.	139.1205	Nicotinamide N-Oxide
47.	140.0769	6-Hydroxynicotinic Acid
48.	141.1000	Methyl Imidazoleacetic Acid
49.	141.9670	Ethanolamine Phosphate
50.	142.1318	O-Phosphoethanolamine
51.	143.1158	5-Hydroxymethyluracil
52.	144.0058	Proline Betaine
53.	144.1475	Methyl-Glutaconic Acid
54.	144.9911	N-Butyrylglycine
55.	145.1313	Caprylic Acid
56.	146.0571	4-Acetamidobutanoate
57.	146.1265	Spermidine
58.	146.9783	Alpha-Ketoglutaric Acid
59.	147.0897	Lysine
60.	147.1236	Glutamine
61.	148.0521	N-Acetylserine
62.	148.1045	Glutamic Acid
63.	148.9860	Citramalate
64.	149.0674	D-2-Hydroxyglutaric Acid
65.	149.1275	Mevalonic Acid
66.	150.0674	L-Methionine
67.	150.1202	3-Methyladenine
68.	151.0523	(S,S)-Tartaric Acid
69.	151.1214	D-Ribose & Isomers
70.	152.0379	Guanine
71.	152.1185	Hydroxy Adenine
72.	153.1372	Xanthine
73.	153.9654	Dopamine
74.	155.0571	2,4-Dihydroxybenzoic Acid
75.	155.1360	Hydroxytyrosol
76.	156.0893	Histidine
77.	157.0937	Orotic Acid
78.	158.0107	3-Methylcrotonylglycine
79.	158.1057	2-Amino-Muconic Acid

80.	158.1643	Tiglylglycine
81.	158.9726	N-Acetylproline
82.	159.0806	Allantoin
83.	159.1247	3-Oxooctanoic Acid C8
84.	160.0704	Isovalerylglycine
85.	161.1066	Methyl Adipic Acid
86.	161.1424	Tryptamine
87.	162.0172	Alpha-Aminoadipate
88.	162.1005	Indole-3-Ethanol
89.	162.1231	L-Carnitine
90.	163.0416	3-Hydroxy-3-Methylglutarate
91.	163.0862	2-Hydroxyadipic Acid
92.	164.9388	Acetylcysteine
93.	165.0651	2,4-Dihydroxypteridine
94.	165.1011	2-Deoxy-D-Glucose
95.	165.1424	Hydroxycinnamic Acid
96.	166.1015	Phenylalanine
97.	167.0441	Methoxyphenylacetic Acid
98.	167.1170	Phenyllactic Acid
99.	169.0490	Uric Acid
100.	169.1515	Pyridoxamine
101.	169.9884	6-Hydroxydopamine
102.	170.1055	1-Methylhistidine
103.	171.1601	Propylthiouracil
104.	172.9832	Decanoic Acid C10:0
105.	173.1657	Glycerol 3-Phosphate
106.	175.1438	Arginine
107.	176.0239	Indoleacetic Acid
108.	176.0977	N-Acetyl-L-Aspartic Acid
109.	176.9839	Ascorbic Acid
110.	177.1244	Serotonin
111.	178.0729	N-Formyl-L-Methionine
112.	179.0121	Cysteinylglycine
113.	179.0959	D-Glucono-1,5-Lactone
114.	180.1708	Hippuric Acid
115.	181.0742	Nicotinuric Acid
116.	181.1340	Glucose
117.	181.9605	Tyrosine
118.	182.0900	Hydroxy-7-Methylguanine
119.	182.9966	Sorbitol
120.	183.1230	Homo(Methyl)Vanillic Acid

121.	185.0359	Dihydroxymandelic Acid
122.	186.2146	Phosphoserine
123.	188.0877	N1-Acetylspermidine
124.	189.0999	Azelaic Acid
125.	189.1391	Cresyl Sulfate
126.	189.1738	Trimethyllysine
127.	190.0631	Kynurenic Acid
128.	190.9976	3-Hydroxysuberic Acid
129.	191.1164	2,6-Diaminoheptanedioate
130.	191.1803	5-Methoxymethyl Adipic Acid
131.	192.9891	5-Hydroxyindoleacetic Acid
132.	193.1639	Citric acid
133.	195.1840	4-Aminohippuric Acid
134.	197.1800	1,3-Dimethyluric Acid
135.	199.1907	Vanillylmandelic Acid
136.	200.2359	O-Phosphothreonine
137.	201.1759	Dodecanoic Acid
138.	202.0289	N-Acetylaminooctanoic Acid
139.	202.2296	Cysteine-S-Sulfate
140.	**203.0655**	**Dimethylarginine/Spermine**
141.	**204.1369**	**L-Acetylcarnitine**
142.	**205.1072**	**L-Tryptophan**
143.	207.1749	Lipoic Acid
144.	211.1308	Glucaric Acid
145.	213.1737	Vanillactic Acid
146.	214.2671	Indoxyl Sulfate
147.	215.1395	Methyl lauric acid
148.	215.2006	Tridecanoic Acid
149.	216.2445	Propenoylcarnitine
150.	217.1707	N-Acetyl-Arginine
151.	219.1879	N-Acetylserotonin
152.	221.1299	5-Hydroxy-Tryptophan
153.	223.1095	L-Cystathionine
154.	224.1981	N-Acetyl-Tyrosine
155.	227.1899	Carnosine
156.	227.9970	Deoxycytidine
157.	228.2325	Deoxy Cytidine
158.	229.1570	Deoxy Uridine
159.	229.2162	Myristic Acid
160.	230.2632	Butenyl Carnitina
161.	235.1837	Methoxytryptophan

162.	237.2041	Phenylalanyl-Alanine
163.	239.2510	5-Oxo-Tetradecadienoic Acid (C14:2)
164.	244.0944	Tiglylcarnitine
165.	245.0972	Uridine
166.	251.0143	Threonylmethionine
167.	255.0092	Histidylvaline
168.	256.2750	Palmitamide
169.	258.2956	Glycerophosphocholine
170.	261.0680	D-Glucose-6-Phosphate
171.	269.1789	Homocystine
172.	269.2235	Inosine
173.	277.0551	6-Phosphogluconic Acid
174.	279.2463	Linolenic C18:3
175.	281.2637	Linoleic Acid C18:2
176.	283.2135	1-Methyladenosine
177.	283.2712	Oleic Acid C18:1
178.	284.3453	Guanosine
179.	285.1831	Xanthosine
180.	285.2742	Stearic Acid C18:0
181.	286.1146	2'-Deoxyguanosine
182.	286.1210	2'-Deoxyguanosine
183.	286.3271	L-Octenoylcarnitine
184.	288.3066	C17 Sphinganine/L-Octanoylcarnitine
185.	299.1274	Benzoil Glucuronide, 7-Etylguanosine
186.	299.2757	Hydroxy Oleic Acid
187.	300.2230	D-Sphingosine
188.	302.5827	Sphinganine
189.	303.2602	Arachidonyl Alanine, Palmitoyl Ethanolamine
190.	304.3177	Pimelylcarnitine
191.	305.1886	Eicosatetraenoic (Arahidonic) Acid C20:4
192.	307.2650	5,11-Eicosatrienoic Acid C20:3
193.	309.1825	2'-Deoxyuridine 5'-Mono-Phos-Phate
194.	309.2370	5,11-Eicosadienoic Acid C20:2
195.	313.2937	Eicosanoic (Arachidic) Acid C20:0
196.	314.2399	9-Decenoylcarnitine
197.	316.2673	Decanoyl Carnitine
198.	317.1349	Etyltestosterone
199.	323.1647	Thymidine 5'-Monophosphate
200.	326.3010	N-Myristoyl Proline
201.	329.2604	Docosahexaenoic Acid (C22:6)
202.	331.0208	Deoxycorticosterone

203.	331.2367	Docosapentaenoic Acid (C22:5)
204.	331.3037	Mg(0:0/16:0/0:0)
205.	332.3492	N-Ethyl Arachidonoyl Amine
206.	333.1640	Hydroxypregnenolone
207.	335.1431	Nicotinamide Mononucleotide
208.	335.2980	Docosatrienoic Acid C22:3
209.	341.2508	Fructose 1,6-Biphosphate
210.	341.3247	9-Hexadecenoylcholine
211.	343.2411	Sucrose
212.	346.0280	Guanosine 3',5'-Cyclic Monophosphate
213.	349.2856	Inosine 5'-Phosphate
214.	353.2868	Prostaglandin E2/D2
215.	355.2959	Mg(0:0/18:2/0:0)
216.	357.2976	Mg(0:0/18:1/0:0)
217.	359.3361	Mg(0:0/18:0/0:0)
218.	363.2400	Cortisol
219.	365.1034	Xanthosine 5'-Monophosphate
220.	365.3012	Tetracosadienoic Acid C24:2
221.	366.0156	Sphingosine-1-P (C17 Base)
222.	367.2655	Tetracosaenoic Acid C24:1
223.	368.4469	Sphinganine-1-P (C17 Base)
224.	369.2677	Tetracosanoic Acid C24:0
225.	369.3722	Lignoceric Acid
226.	370.2295	Tetradecenoylcarnitine
227.	371.2955	Thromboxane B2
228.	372.3682	Tetradecanoylcarnitine
229.	375.2733	3b-Hydroxy-5-Cholenoic Acid
230.	377.1642	Riboflavin
231.	379.3181	MG 20:4
232.	381.3197	MG(20:3)
233.	383.2258	MG(20:2)
234.	385.2269	s-(5'-Adenosyl)-L-Homocysteine/ Vitamin D3 (Deydrocholesterol)
235.	385.3121	Mycolipanolic Acid (C24:0)/MG(20:2)
236.	387.2777	Tricaproin (C6:0)
237.	387.3445	Cholesterol
238.	388.2756	2'-DEOXYCYTIDINE 5'-DIPHOSPHATE
239.	391.3077	12-Ketodeoxycholic Acid
240.	393.2629	Hyodeoxycholic Acid
241.	394.3731	C19 Sphingosine-1-Phosphate
242.	396.3541	Sphingosine 1 Phosphate
243.	397.2870	Delta Tocotrienol

244.	397.3142	Pregnenolone Sulfate
245.	399.3296	N-Palmitoyltryptamine
246.	400.4005	Palmitoyl Carnitine, S-Adenosyl Metionine
247.	405.2479	Cortisol 21-Acetate
248.	409.2555	Ursocholic/Muricholic Acid Acid
249.	413.2947	25-Hydroxyvitamin D2
250.	414.2581	Heptadecanoyl Carnitine
251.	415.2573	Ascorbyl Palmitate
252.	419.2139	Palmitoyl Glucuronide
253.	425.2370	Alpha-Tocotrienol
254.	426.3780	O-Oleoylcarnitine
255.	428.2863	Stearoylcarnitine, ADP
256.	429.3354	Cholesteryl Acetate
257.	429.3734	Colesteryl Acetate, Lactoseaide Glucuronide
258.	440.3825	3-Hydroxylinoleoylcarnitine
259.	443.3562	Ascorbyl Stearate
260.	445.3450	1α,24,25-Trihydroxyvitamin D2
261.	456.3772	Arachidyl Carnitine
262.	458.3474	N-Docosahexaenoyl Glutamic Acid
263.	463.3265	Arahidonoyl Serotonin
264.	468.3342	LPC(14:0)
265.	471.3792	Tricaprylin (C8:0)
266.	472.3516	Cervonyl Carnitine
267.	478.3263	LPE 18:2
268.	482.4048	LPC 15:0
269.	482.4299	LPC 15:1
270.	484.4097	O-Behenoylcarnitine
271.	485.2951	Uridine 5'-Triphosphate
272.	485.3853	LPA(22:5)
273.	487.3841	LPA(22:5)
274.	489.3679	Cytidine 5'-Diphosphocholine
275.	494.3487	LPC (16:1)
276.	496.3751	LPC (16:0)
277.	502.3993	LPE(20:4)
278.	504.2933	LPE(20:3)
279.	507.3401	Oleyl Palmitate & Isomers C34H66O2
280.	509.4034	Stearyl Palmitate
281.	516.3799	LPC(18:4)
282.	518.3176	LPC(18:3)
283.	**520.3663**	**LPC(18:2)**
284.	522.3823	LPC(18:1)

285.	523.8918	Guanosine Triphosphate
286.	524.3987	LPC(18:0)
287.	526.4304	LPS(18:0)
288.	529.3151	Linolenyl Oleate
289.	531.4349	Linolenyl Stearate
290.	537.4373	Stearyl Stearate & Isomers
291.	540.1825	Hexacosanoyl Carnitine
292.	540.4095	Cer(D18:0/16:0)
293.	542.3503	LPC(20:5)
294.	544.3668	LPC (20:4)
295.	546.4028	LPC (20:3)
296.	548.0824	LPC (20:2)

*all proteogenic amino acids (AA) identified in this study are L amino acids (because all identifications were performed on human plasma samples), although the method used does not discriminate between L and D series.
